# Dnq-unet: a two-level fusion framework for few-shot domain adaptation in cervical cancer CTV segmentation

**DOI:** 10.1007/s12194-026-01072-8

**Published:** 2026-06-03

**Authors:** Boying Li, Yihui Liu, Yanfei Pan, Yuhui Zhang

**Affiliations:** Department of Radiotherapy, People’s Hospital of Ningxia Hui Autonomous Region, Yinchuan, 750002 Ningxia China

**Keywords:** Cervical cancer radiotherapy, Clinical target volume segmentation, Few-shot domain adaptation, Hierarchical feature fusion, Deep learning

## Abstract

To develop and validate a two-level hierarchical fusion architecture enabling efficient few-shot domain adaptation for cervical cancer clinical target volume (CTV) auto-segmentation across different institutional imaging and contouring settings. We propose DNQ-UNet, a dual-encoder architecture with two fusion levels: spatially adaptive normalization (SPADE) for shallow appearance alignment and cross-attention for deep semantic transfer. A progressive two-stage training strategy with grouped learning rates was employed. Few-shot adaptation used 3, 5, 10, 20, 40, and 60 fine-tuning cases, and additional 3D fine-tuning baselines, including nnU-Net V2, SwinUNETR, and MedNeXt, were evaluated under identical target-domain test settings. Ablation studies assessed each fusion component. On the target-domain test set, zero-shot DSC was *0.8040± 0.0549*. With 3, 5, and 10 fine-tuning cases, DSC improved to *0.818± 0.073*, *0.830± 0.050*, and *0.838± 0.056*, respectively. DNQ-UNet achieved the highest DSC among the compared 3D baselines at 5-shot (0.830 vs. 0.805–0.817) and 10-shot (0.838 vs. 0.823–0.833) settings. Ablation showed significant DSC reductions after removing either fusion level or using a single U-Net (all *p<0.001*). The proposed framework enables efficient few-shot domain adaptation for cervical cancer CTV segmentation using 5–10 annotated cases, providing a locally adapted contour-initialization approach that may reduce annotation and repeated correction burden during cross-institutional deployment.

**Supplementary Information:**

The online version contains supplementary material available at https://doi.org/10.1007/s12194-026-01072-8.

## Introduction

Cervical cancer remains a major global health burden, with 662,301 new cases and 348,874 deaths estimated worldwide in 2022. Radiotherapy is a cornerstone of treatment for cervical cancer, and accurate delineation of the clinical target volume (CTV) is essential for modern intensity-modulated and image-guided radiotherapy workflows. However, manual contouring remains time-consuming and subject to substantial inter-observer variability, which limits consistency and efficiency in routine practice [1–5].

Deep learning-based auto-segmentation has shown promising performance for cervical cancer CTV delineation, especially with U-Net-based architectures. Recent studies have reported strong results on single-institution datasets and in external validation settings [6–11]. Nevertheless, cross-domain performance degradation remains a major barrier to clinical deployment because models trained at one institution may underperform when applied to data from other centers with different CT scanners, acquisition protocols, slice thicknesses, and contouring conventions [12–14].

Domain adaptation techniques such as adversarial feature alignment, transfer learning, and meta-learning have been investigated to address this problem [15–18]. In practice, however, large annotated target-domain datasets are often unavailable. Few-shot domain adaptation is therefore particularly attractive for institutions with limited annotated target-domain data, where large-scale expert re-annotation is difficult to obtain.

We selected cervical cancer CTV as the target task because it is a high-volume radiotherapy workflow in our institution and region, and because CTV delineation is clinically important for target coverage and organ-at-risk sparing. Although cervical CTV is not the only radiotherapy structure affected by domain shift, its boundaries at the uterine fundus, parametrial margins, cervicovaginal junction, and postoperative or anatomically variable regions are influenced by local imaging protocols and contouring conventions. Thus, cervical cancer CTV provides a practical clinical setting for evaluating few-shot domain adaptation, while the proposed framework can be further validated in other tumor sites and organ-at-risk segmentation tasks.

Although existing few-shot segmentation and domain adaptation methods have reduced the need for target-domain annotation, many are designed for episodic learning, prompt-based segmentation, or 2D benchmark settings, which may not directly match 3D radiotherapy CTV deployment. Compared with episodic support-set methods such as ALPNet, UniverSeg, and ADNet [19–21], which typically require episodic training or support information at inference, DNQ-UNet follows a pre-training/fine-tuning paradigm and integrates domain adaptation into the architecture through hierarchical fusion. This design is aligned with radiotherapy deployment, where a fixed 3D model is adapted once to local cases and then used for contour initialization in routine planning.

We propose DNQ-UNet, a hierarchical two-level fusion framework for efficient few-shot domain adaptation in cervical cancer CTV segmentation. The architecture decouples prior extraction from fine segmentation through a dual-encoder design: the first U-Net extracts domain-adaptive priors and multi-scale guidance features, while the second U-Net performs high-resolution segmentation. Two complementary fusion mechanisms operate at different granularities: spatially adaptive normalization at shallow layers for appearance alignment, and cross-attention at deep layers for semantic knowledge transfer [22–25]. Progressive training with grouped differential learning rates is used to promote stable adaptation while preserving the segmentation capability learned in the source domain.

## Materials and methods

### Dataset and preprocessing

The source domain comprised 508 CT cases from a single high-volume institution, acquired using a GE Discovery CT590 RT scanner for spiral abdominal/pelvic radiotherapy-planning CT. The nominal voxel spacing was $1.0 \times 1.0 \times 3.0~\textrm{mm}^3$, and the cohort included mixed contrast-enhanced and non-contrast scans. All cases were annotated with cervical CTV contours according to Radiation Therapy Oncology Group (RTOG) guidance. The dataset was split into training (90%) and validation (10%) subsets using a fixed random seed ($\textrm{seed}=42$). Key preprocessing statistics derived from the source-domain training set were as follows: resampled target voxel spacing, $1.171875 \times 1.171875 \times 5.0~\textrm{mm}^3$; median bounding box, $167.6 \times 134.8 \times 220.0~\textrm{mm}$; and foreground intensity, *3.12 ± 86.78* HU after 0.5–99.5 percentile clipping to *[-1024, 278]* HU.

For source-domain evaluation, a separate test set of 102 CT cases from the same institution was collected and completely excluded from training and validation. For target-domain adaptation, an external target-domain cohort of 80 CT cases was collected from Ningxia People’s Hospital. The target scans were acquired using a Philips Brilliance CT Big Bore scanner for spiral abdominal/pelvic radiotherapy-planning CT, with nominal voxel spacing of $1.172 \times 1.172 \times 5.0~\textrm{mm}^3$ and mixed contrast-enhanced and non-contrast scans. Target CTV contours were delineated according to local institutional protocols by radiation oncologists with 5–15 years of experience. Sixty cases formed the maximum adaptation pool, from which progressively larger few-shot fine-tuning subsets containing 3, 5, 10, 20, 40, and 60 cases were prepared using the same case subsets for all compared methods. An independent held-out target-domain test set of 20 cases, also excluded from training and validation, was used for final few-shot evaluation.

**Fig. 1:**
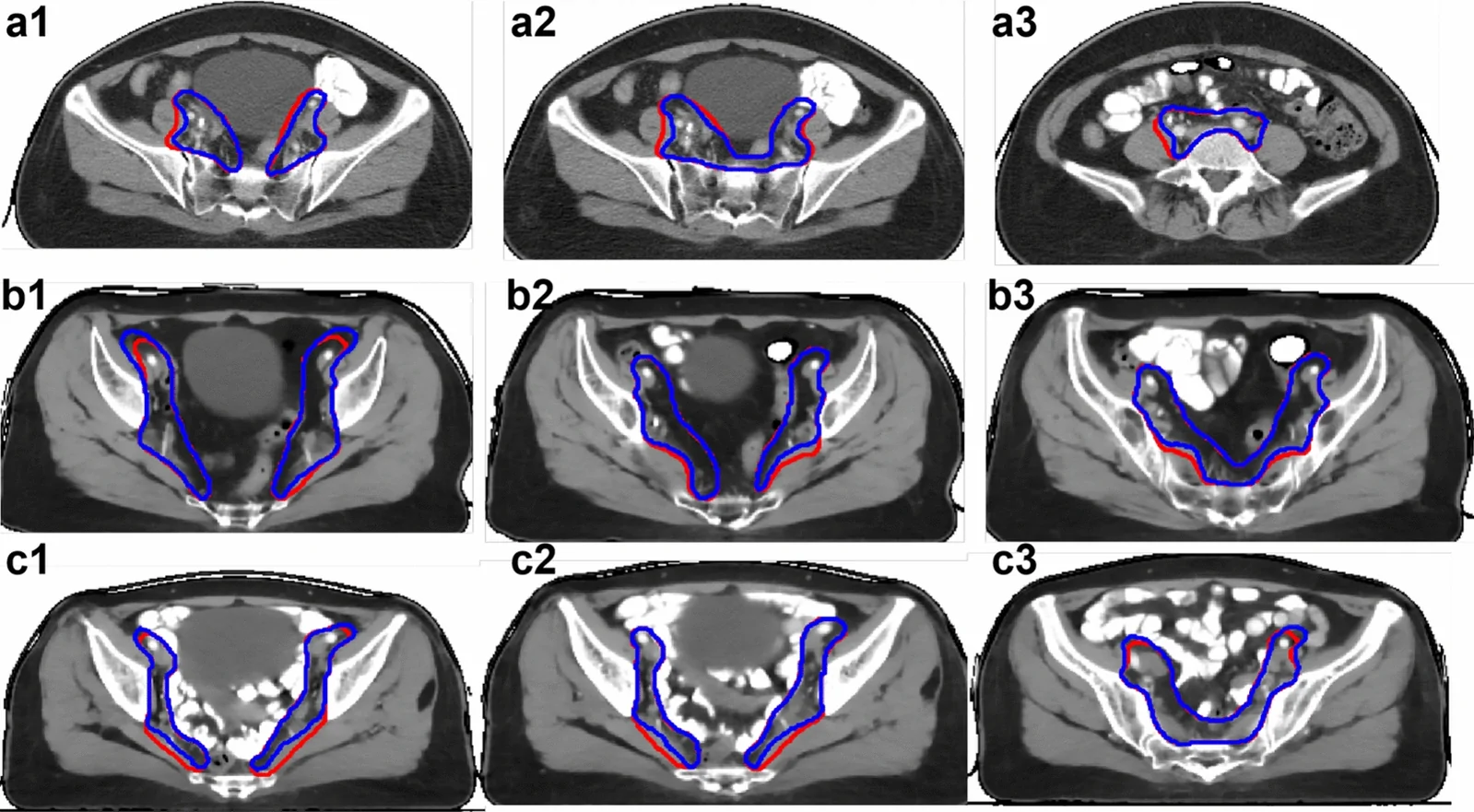
Representative cross-domain contouring differences. Axial CT slices show CTV contours from source-domain physicians (red) and target-domain physicians (blue) on shared cases. Differences are visible around the uterine fundus, parametrial margins, and cervicovaginal junction. Annotation review identified 46 discrepant slices across five shared cases, with a maximum $1-\textrm{IoU}$ of 0.231, illustrating institutional contouring variability in addition to imaging-protocol differences

To clarify the acquisition characteristics of the two domains, scanner-related information was extracted from original CT DICOM headers and treatment-planning records (Table 1). The source and target domains were acquired at different institutions using different CT scanners and local planning workflows: GE Discovery CT590 RT for the source domain and Philips Brilliance CT Big Bore for the target domain. Original acquisition parameters, including CT scanner, acquisition protocol, voxel spacing, and contrast-enhancement status, were distinguished from the resampled spacing used for model training. Contouring-related information, including contouring guideline, annotator background, and review procedure, was obtained from RT structure files, treatment-planning-system records, and institutional contouring protocols. Five target-domain CT cases were independently re-contoured by physicians following source-domain and target-domain contouring conventions. Inter-institutional annotation differences were quantified on five shared cases, revealing 46 discrepant slices with a maximum $1-\textrm{IoU}$ of 0.231, mainly around the uterine fundus, parametrial margins, and cervicovaginal junction. Representative inter-institutional contouring differences are illustrated in Fig. 1.

Preprocessing followed the nnU-Net v2 workflow [26]: (1) resampling to a target spacing of $1.171875 \times 1.171875 \times 5.0~\textrm{mm}^3$ using trilinear interpolation for CT images and nearest-neighbor interpolation for segmentation labels; (2) *z*-score intensity normalization using source-domain foreground statistics (*μ =3.12*, *σ =86.78*); (3) CT intensities were clipped to the foreground 0.5th–99.5th percentile range *[-1024,278]* HU before normalization; (4) mapping all non-zero CTV label values to 1 as the foreground class; and (5) foreground-oversampled patch extraction with patch size $160 \times 160 \times 64$ voxels and foreground sampling probability 33%, with boundary padding when required.

**Table 1:** Summary of source- and target-domain imaging and contouring characteristics

Characteristic	Source domain	Target domain
Dataset use	508 training/validation cases; 102 independent source-domain test cases	80 external cases: 60-case adaptation pool; 20 independent target-domain test cases
Institutional setting	Single source institution	Ningxia People’s Hospital; local target-domain planning workflow
Scanner and protocol	GE Discovery CT590 RT; spiral abdominal/pelvic radiotherapy-planning CT; mixed contrast-enhanced and non-contrast scans	Philips Brilliance CT Big Bore; spiral abdominal/pelvic radiotherapy-planning CT; mixed contrast-enhanced and non-contrast scans
Nominal voxel spacing	$1.0\times 1.0\times 3.0~\textrm{mm}^3$	$1.172\times 1.172\times 5.0~\textrm{mm}^3$
Contouring protocol	CTV delineated according to RTOG guidance	CTV delineated according to local institutional protocols by radiation oncologists with 5–15 years of experience
Training preprocessing	Resampled to $1.171875\times 1.171875\times 5.0~\textrm{mm}^3$ with identical intensity normalization	Same preprocessing and resampling as the source domain

### Network architecture

**Fig. 2:**
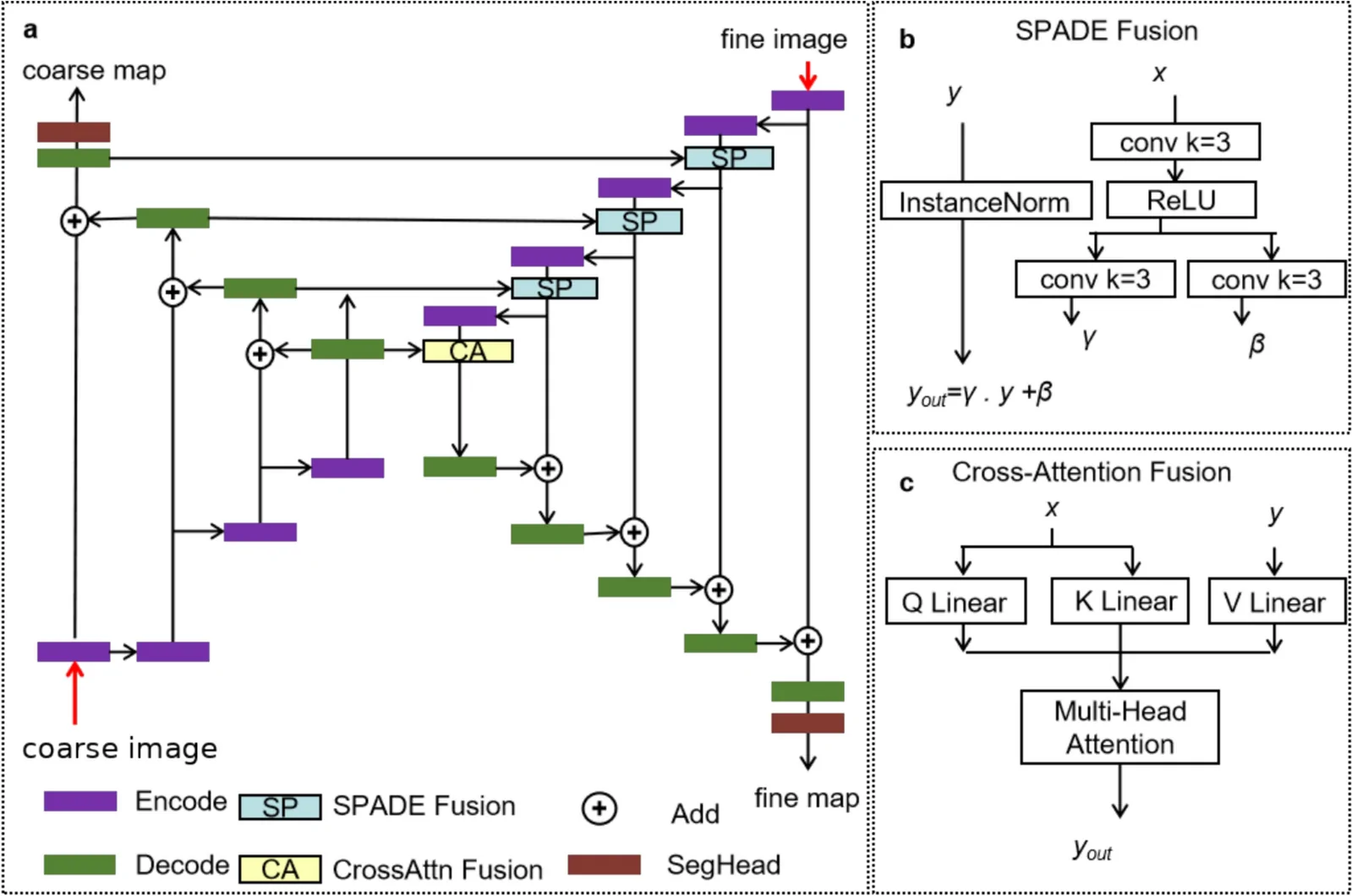
DNQ-UNet architecture overview. **a** Overall framework showing the dual-encoder design with two-level hierarchical fusion. **b** Level 1 fusion: SPADE conditional normalization for appearance alignment at shallow layers ($S_1$–$S_3$). **c** Level 2 fusion: cross-attention for semantic knowledge transfer at the deep layer ($S_4$)

Figure 2 illustrates the DNQ-UNet dual-encoder architecture, in which two complementary fusion levels operate at different spatial scales and semantic depths.

#### First U-Net (prior extractor)

The first U-Net uses a 4-level encoder with channel progression $32 \rightarrow 64 \rightarrow 128 \rightarrow 256$ and processes a spatially downsampled input of $80 \times 80 \times 32$ voxels. Anisotropic pooling kernels $\{(2,2,1),(2,2,1),(2,2,2),(2,2,2)\}$ are employed to preserve axial resolution in early layers and to accommodate the typical anisotropy of pelvic CT data. A nested decoder with skip connections produces decoder features $D_0$–$D_3$ at multiple resolutions, together with a coarse segmentation output.

#### Second U-Net (fine segmentation engine)

The second U-Net uses a 5-level encoder with channel progression $32 \rightarrow 64 \rightarrow 128 \rightarrow 256 \rightarrow 512$ and processes the full-resolution input of $160 \times 160 \times 64$ voxels. The input is the original single-channel CT image. Multi-scale prior information extracted by the first U-Net is injected into the second U-Net only through the two fusion pathways described below.

### Two-level fusion mechanism

The two fusion levels operate at different spatial scales and with different mechanisms, thereby providing complementary forms of prior transfer from the first U-Net to the second U-Net. Shallow layers receive spatially adaptive normalization cues, whereas the deepest fusion layer receives semantic information through cross-attention.

#### Level 1: SPADE conditional normalization

At the three shallow encoder levels of the second U-Net ($S_1$, $S_2$, and $S_3$), spatially adaptive denormalization (SPADE) is applied using the corresponding decoder features from the first U-Net as spatial conditioning signals [22]. In contrast to batch normalization [27], SPADE produces spatially varying normalization parameters after instance normalization [28]:

1 $$\begin{aligned} S_i' = \gamma _i \odot \operatorname {InstanceNorm}(S_i) + \beta _i , \end{aligned}$$

where

$$ (\gamma _i,\beta _i)=f_{\textrm{SPADE}}(D_{\textrm{guide}}), $$

with mapping $D_3 \rightarrow S_1$, $D_2 \rightarrow S_2$, and $D_1 \rightarrow S_3$. When needed, the guide features are trilinearly interpolated to match the spatial size of $S_i$. The SPADE modules use guide-channel/feature-channel configurations of 32/32 for $S_1$, 32/64 for $S_2$, and 64/128 for $S_3$.

For stable optimization, the SPADE layers were initialized close to the identity transformation: the gamma-convolution weights were set to 0 with bias 1, and the beta-convolution weights were set to 0 with bias 0. SPADE was preferred over global attention at shallow levels because the large feature maps would make full attention computationally expensive.

#### Level 2: cross-attention knowledge transfer

At the deepest fusion level ($S_4$), global 3D multi-head cross-attention is applied [23, 24]. The second U-Net features act as queries, while the first U-Net decoder features act as keys and values. Specifically, $S_4$ has spatial size $10 \times 10 \times 16$ (1600 spatial tokens), whereas $D_0$ has spatial size $5 \times 5 \times 8$ (200 tokens) and is interpolated to the same resolution before attention:

2 $$\begin{aligned} \operatorname {Attention}(\textbf{Q},\textbf{K},\textbf{V})= \operatorname {softmax}\!\left( \frac{\textbf{Q}\textbf{K}^{\textsf{T}}}{\sqrt{d_k}}\right) \textbf{V}, \end{aligned}$$

3 $$\begin{aligned} S_4' = S_4 + \operatorname {Linear}\!\left( \operatorname {Attention}(\textbf{Q},\textbf{K},\textbf{V})\right) , \end{aligned}$$

Here, $\textbf{Q}=\operatorname {LayerNorm}(S_4)W_Q$, $\textbf{K}=\operatorname {LayerNorm}(D_0)W_K$, and $\textbf{V}=\operatorname {LayerNorm}(D_0)W_V$. The attention module uses 4 heads with $d_k=64$. The residual connection allows the second U-Net to incorporate first U-Net knowledge selectively rather than forcing dependence on it.

The total parameter count is approximately 29.1 million, including approximately 7.1 million parameters in the first U-Net, 21.2 million in the second U-Net, and 0.68 million in the fusion modules.

### Training strategy

All training and inference were performed on three-dimensional CT volumes rather than two-dimensional slice images. The network used 3D convolutional operations with volumetric patches of $160 \times 160 \times 64$ voxels. The main training configuration is summarized in Table 2.

**Table 2:** Training configuration summary

Phase	Epochs	Base learning rate	Batch size	Trainable modules
Stage 1 pre-training	500	$1\times 10^{-3}$	4	First U-Net (*∼ *7.1M)
Stage 2 joint training	1000	$3\times 10^{-4}$	4	All modules (*∼ *29.1M)
Few-shot fine-tuning	300	$3\times 10^{-5}$	4	All modules (*∼ *29.1M)

All phases used the AdamW optimizer with weight decay of $1\times 10^{-5}$, PolyLR scheduling with exponent 0.9, gradient clipping with maximum norm 12.0, and FP16 mixed precision. During Stage 2 and few-shot fine-tuning, grouped learning rates were used, with the first U-Net updated at 0.1 times the base learning rate. The epoch numbers were fixed before final test-set evaluation based on validation convergence, and final checkpoints were selected according to validation pseudo-Dice

#### Stage 1 pre-training

The first U-Net was first trained alone for 500 epochs on downsampled inputs using a combination of cross-entropy loss and Dice loss [29]:

4 $$\begin{aligned} L_{\textrm{Stage1}} = L_{\textrm{CE}}(O_{\textrm{coarse}},Y) + L_{\textrm{Dice}}(O_{\textrm{coarse}},Y). \end{aligned}$$

Optimization used AdamW [30] with learning rate $1\times 10^{-3}$, weight decay $1\times 10^{-5}$, PolyLR scheduling (exponent 0.9), gradient clipping with maximum norm 12, and mixed-precision training (FP16).

#### Stage 2 joint training

The full architecture was then trained for 1000 epochs using grouped differential learning rates to limit catastrophic forgetting. The learning rates were set to $3\times 10^{-5}$ for the first U-Net (0.1 times the base rate), $3\times 10^{-4}$ for the second U-Net, and $3\times 10^{-4}$ for the fusion modules. The total loss combined fine supervision, coarse supervision, and prediction consistency:

5 $$\begin{aligned} L_{\textrm{total}} = L_{\textrm{fine}} + 0.3\,L_{\textrm{coarse}} + 0.1\,L_{\textrm{consistency}}, \end{aligned}$$

where

$$ L_{\textrm{consistency}} = \operatorname {MSE}\!\left( \operatorname {upsample}(P_{\textrm{coarse}}), P_{\textrm{fine}}\right) . $$

The consistency term encourages agreement between coarse and fine predictions across scales. The weights in Eq. (5) were determined by a preliminary grid search using source-domain validation performance and training stability. The fine segmentation loss was assigned the primary weight because it corresponds to the final clinical output. The auxiliary coarse loss was given a lower weight to maintain prior-extractor supervision without dominating optimization, and the consistency term was assigned a small weight to avoid over-regularization. The independent test sets were not used for hyperparameter selection.

#### Few-shot fine-tuning

Target-domain adaptation started from the Stage 2 checkpoint and was performed for 300 epochs with a base learning rate of $3\times 10^{-5}$. The same grouped learning-rate strategy was maintained during fine-tuning. For each few-shot setting, cases were randomly selected with seed 42 and split into training and validation subsets at an 80/20 ratio when checkpoint selection was required. The epoch numbers were fixed before final test-set evaluation. Stage 1 and Stage 2 used fixed source-domain training budgets, whereas the 300-epoch few-shot schedule was chosen because validation curves plateaued within this range and longer training increased the risk of overfitting on small target-domain subsets. Final checkpoints were selected according to validation pseudo-Dice rather than target test-set performance.

### Experiments and evaluation

Three main experiments were performed.

#### Experiment 1: few-shot adaptation

Target-domain fine-tuning was evaluated using 3, 5, 10, 20, 40, and 60 adaptation cases. The primary end point was the Dice similarity coefficient (DSC); secondary end points were 95% Hausdorff distance (HD95), sensitivity, specificity, and intersection over union (IoU). One-way analysis of variance (ANOVA) with Bonferroni correction was used for group comparison.

#### Experiment 2: comparison with 3D fine-tuning baselines

To compare DNQ-UNet with practical few-shot domain-adaptation baselines, nnU-Net V2 [26], SwinUNETR [31], and MedNeXt [32] were pre-trained on the same 508 source-domain cases and fine-tuned on the same 3-, 5-, and 10-case target-domain subsets for 300 epochs. All methods used the same independent 20-case target-domain test set. Methods that require per-inference support sets, episodic meta-learning, manual prompts, or additional foundation-model pretraining, such as ALPNet, UniverSeg, and self-supervised supervoxel-based approaches [19–21], were discussed qualitatively rather than used as direct 3D baselines.

#### Experiment 3: ablation study

Following the single-variable principle, four configurations were evaluated: (A) full model, (B) without Level 2 cross-attention, (C) without Level 1 SPADE, and (D) single U-Net baseline. Each configuration was trained from scratch on the source domain with identical hyperparameters. Paired *t* tests against the full model were performed with Bonferroni correction ($\alpha _{\textrm{corr}}=0.0167$, *k=3*).

Inference used a sliding-window strategy with patch size $160 \times 160 \times 64$, 50% overlap, and Gaussian importance weighting. Validation checkpoints were selected according to the pseudo-Dice criterion used in nnU-Net v2 [26]. All metrics were computed on binary masks at the target spacing of $1.171875 \times 1.171875 \times 5.0~\textrm{mm}^3$.

## Results

### Few-shot adaptation performance

As a source-domain sanity check, DNQ-UNet retained baseline performance comparable to nnU-Net V2 (Table S1); therefore, the primary analyses focused on target-domain few-shot adaptation and ablation. DNQ-UNet demonstrated clear gains with few-shot target-domain adaptation compared with zero-shot transfer (Table 3). Without any fine-tuning, the source-domain model achieved DSC of *0.804± 0.055* on the target domain, indicating a pronounced domain gap. With 3 and 5 fine-tuning cases, DSC improved to *0.818± 0.073* and *0.830± 0.050*, respectively. With only 10 fine-tuning cases, DSC improved to *0.838± 0.056*, and IoU improved from *0.676± 0.072* to *0.725± 0.077*.

**Fig. 3:**
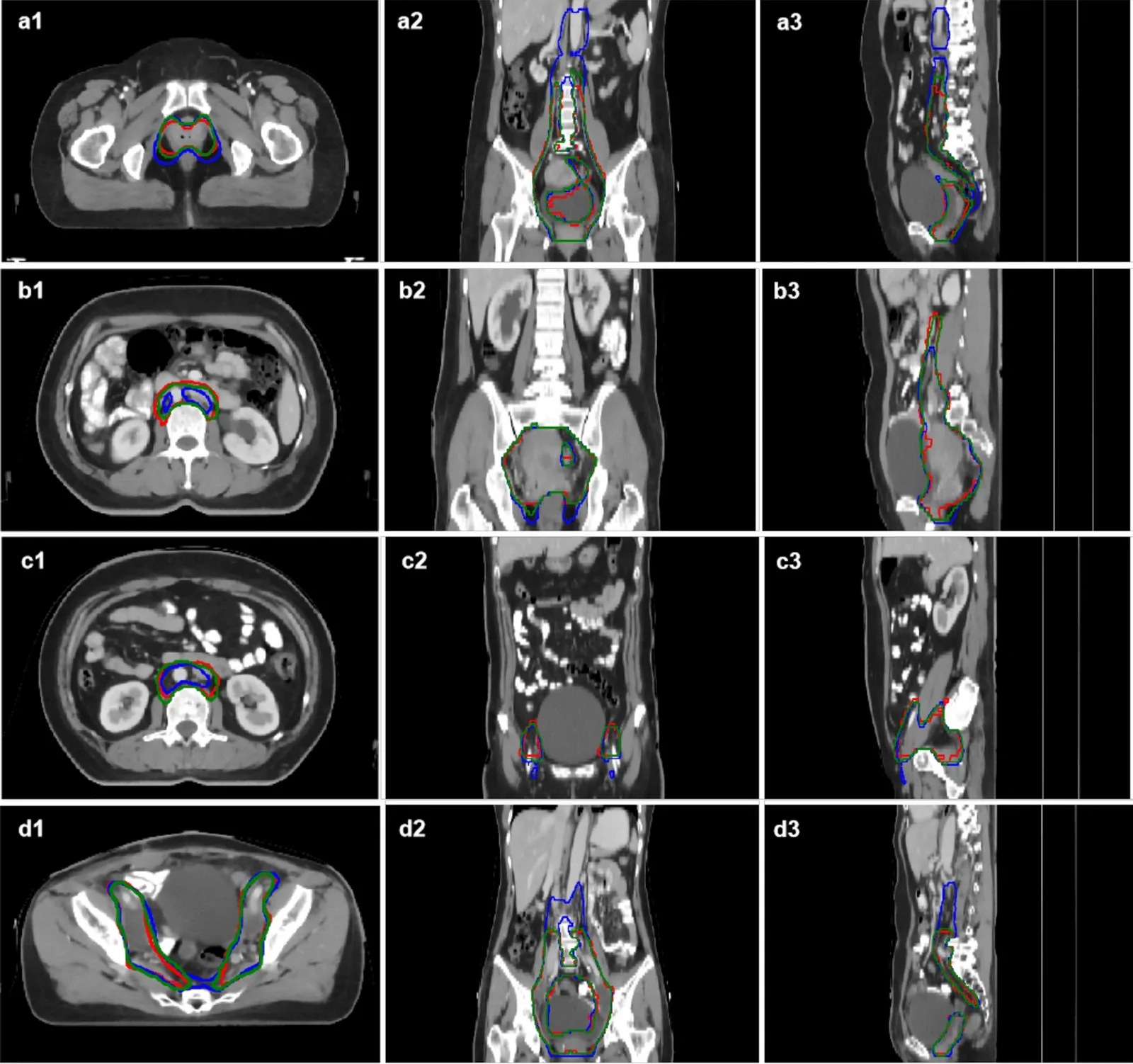
Visual comparison of segmentation results between DNQ-UNet and nnUNetV2 on four representative test cases. Rows **a**–**d** correspond to four different patients. Columns 1–3 show axial, coronal, and sagittal views, respectively. Contour overlays are as follows: red, ground truth; green, DNQ-UNet prediction; blue, nnUNetV2 prediction. DNQ-UNet demonstrates improved boundary delineation and reduced under-segmentation, particularly at the uterine–cervical junction and parametrial boundaries

The 5-shot result already recovered most of the 10-shot improvement, while the 10- and 20-case settings achieved the same mean DSC (0.838). These results suggest that approximately 5–10 annotated cases can provide effective adaptation in this dataset, with 10 cases providing the most stable balance between DSC and boundary accuracy (Fig. 3). At 3-shot, the elevated HD95 (*34.01± 30.54* mm) and high sensitivity (*0.902± 0.037*) indicated boundary instability in the extremely low-data setting; this instability decreased at 5-shot and 10-shot. The 40- and 60-case settings showed slightly lower DSC, suggesting diminishing returns and possible overfitting with larger fine-tuning sets. Specificity remained near ceiling across all settings (mean 0.9978–0.9989).

**Fig. 4:**
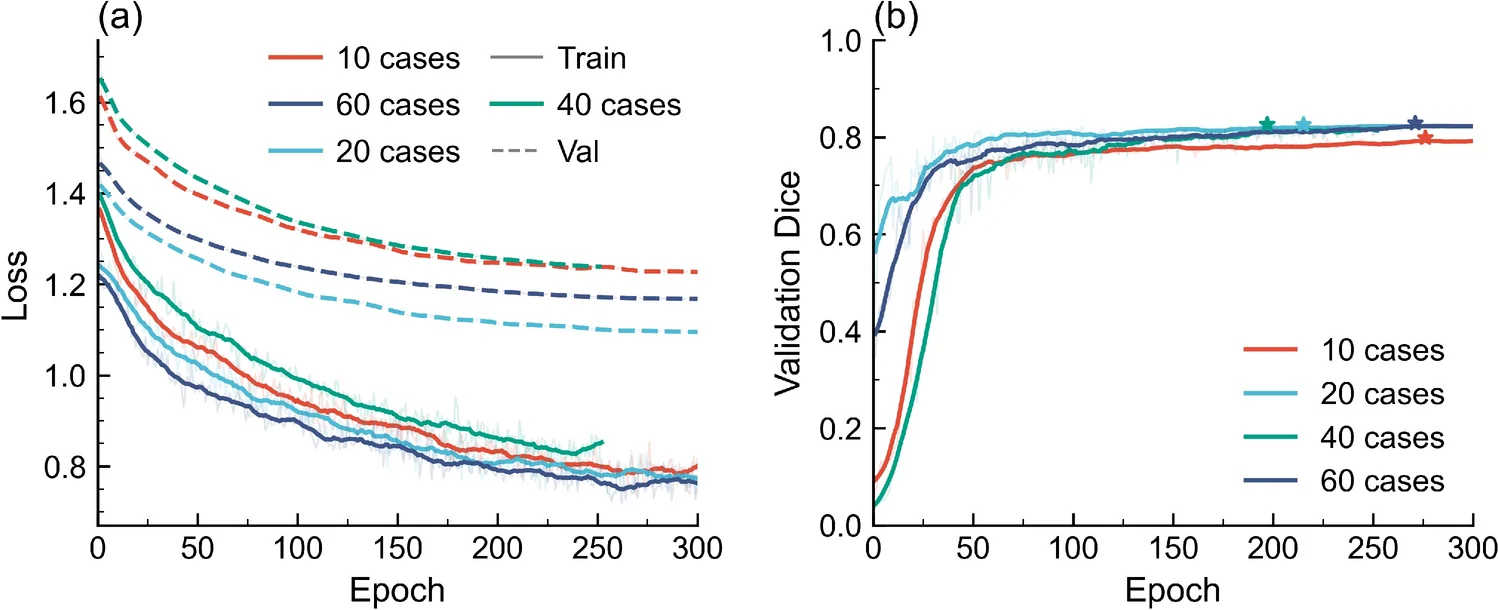
Training curves for the few-shot fine-tuning experiment. **A** Training and validation loss across 300 epochs for the four fine-tuning cohorts (10, 20, 40, and 60 cases). Solid lines indicate training loss and dashed lines indicate validation loss. **B** Validation DSC across epochs for the same cohorts. Stars mark the epoch at which the best validation DSC was achieved for each cohort: 10 cases, 0.799 at epoch 276; 20 cases, 0.826 at epoch 215; 40 cases, 0.827 at epoch 197; and 60 cases, 0.829 at epoch 271

Figure 4 shows the training convergence analysis for the original 10-, 20-, 40-, and 60-case cohorts. The 10-case cohort achieved the best validation DSC (0.799) at epoch 276, whereas the 60-case cohort achieved the best validation DSC (0.829) at epoch 271. Case-wise review indicated that zero-shot transfer was especially weak for several target-domain cases and that these difficult cases improved substantially after fine-tuning, consistent with a systematic distribution shift between the source and target domains.

### Comparison with 3D fine-tuning baselines

Table 4 summarizes the comparison with additional 3D architectures under identical few-shot settings. All methods showed similar zero-shot DSC values (0.793–0.804), confirming comparable target-domain degradation before adaptation. DNQ-UNet achieved the highest DSC in the 5-shot setting (*0.830± 0.050*), exceeding nnU-Net V2 by 0.025, SwinUNETR by 0.016, and MedNeXt by 0.013. At 10-shot, DNQ-UNet again achieved the highest DSC (*0.838± 0.056*) and showed the largest DSC gain from zero-shot to 10-shot adaptation (+0.034). These comparisons indicate that the proposed hierarchical fusion design provides additional sample efficiency beyond conventional full-network fine-tuning of strong 3D segmentation backbones.

### Ablation study

The ablation study on the 102 source-domain test cases quantified the contribution of each fusion component (Table 5). The full model achieved DSC of *0.8502± 0.0454*. Removing Level 2 cross-attention reduced DSC to *0.8356± 0.0474* (*p<0.001*, Cohen’s *d=0.841*), indicating its contribution to semantic refinement. Removing Level 1 SPADE reduced DSC to *0.8380± 0.0453* (*p<0.001*, Cohen’s *d=0.962*), indicating the importance of spatially adaptive appearance alignment.

The single U-Net baseline, which removed all guidance from the first U-Net, achieved DSC of *0.8356± 0.0479*, representing a decrease of 0.0146 from the full model (*p<0.001*, Cohen’s *d=0.971*). All ablation variants showed statistically significant reductions in DSC after Bonferroni correction.

### Qualitative assessment

Representative shared cases illustrating source- and target-domain contouring differences are shown in Fig. 1. Qualitative comparisons between DNQ-UNet and nnUNetV2 are shown in Fig. 3. DNQ-UNet predictions more closely matched the expert reference contours in diverse anatomical settings, particularly at challenging boundaries such as the uterine–cervical junction and parametrial extension. Representative cases showed robust behavior across large target volumes, post-surgical anatomy, and complex pelvic configurations. Remaining errors mainly consisted of mild under-segmentation at the uterine–cervical junction and occasional over-segmentation into the adjacent bladder wall.

**Table 3:** Few-shot adaptation performance on the independent target-domain test set (*n=20*)

Setting	Cases	DSC	IoU	HD95 (mm)	Sens	Spec. (mean)
Zero-shot	0	*0.804± 0.055*	*0.676± 0.072*	*15.73± 15.83*	*0.878± 0.058*	0.9982
3-shot	3	*0.818± 0.073*	*0.692± 0.088*	*34.01± 30.54*	*0.902± 0.037*	0.9978
5-shot	5	*0.830± 0.050*	*0.713± 0.071*	*18.71± 23.37*	*0.858± 0.075*	0.9989
10-shot	10	*0.838± 0.056*	*0.725± 0.077*	*12.42± 13.39*	*0.861± 0.063*	0.9989
20-shot	20	*0.838± 0.055*	*0.725± 0.076*	*15.89± 13.44*	*0.877± 0.059*	0.9988
40-shot	40	*0.831± 0.051*	*0.714± 0.072*	*24.56± 26.71*	*0.880± 0.062*	0.9987
60-shot	60	*0.835± 0.049*	*0.719± 0.070*	*20.50± 24.43*	*0.885± 0.057*	0.9987

Values for DSC, IoU, HD95, and sensitivity are reported as mean±SD. Specificity is reported as mean only because it was near ceiling across all settings and was less discriminative in background-dominated volumetric segmentation. The elevated 3-shot HD95 reflects boundary instability in the extremely low-data setting, which decreased at 5-shot and 10-shot. DSC, Dice similarity coefficient; IoU, intersection over union; HD95, 95% Hausdorff distance

**Table 4:** Comparison of 3D fine-tuning baselines on cervical CTV segmentation

Method	Strategy	Source-domain	0-shot	3-shot	5-shot	10-shot
nnU-Net V2 [26]	Full fine-tune	*0.844± 0.051*	*0.800± 0.055*	*0.815± 0.060*	*0.805± 0.073*	*0.833± 0.063*
SwinUNETR [31]	Full fine-tune	*0.833± 0.046*	*0.799± 0.055*	*0.811± 0.067*	*0.814± 0.070*	*0.826± 0.058*
MedNeXt [32]	Full fine-tune	*0.838± 0.047*	*0.793± 0.051*	*0.817± 0.066*	*0.817± 0.070*	*0.823± 0.065*
DNQ-UNet (ours)	Hierarchical DA	*0.850± 0.045*	*0.804± 0.055*	*0.818± 0.073*	*0.830± 0.050*	*0.838± 0.056*

All methods were pre-trained on the same 508 source cases, fine-tuned on the same *n*-shot target cases, and tested on the 102 source-domain and 20 target-domain test cases. Values are DSC mean±SD

**Table 5:** Ablation study results on the source-domain test set (*n=102*)

Configuration	Level 1	Level 2	DSC	HD95 (mm)	*Δ *DSC vs. full	Cohen’s *d*	*p* value
Full model	*\checkmark *	*\checkmark *	*0.8502± 0.0454*	*11.15± 26.37*	–	–	–
w/o Level 2	*\checkmark *	*× *	*0.8356± 0.0474*	*14.09± 40.28*	*-0.0146*	0.841	*<0.001*
w/o Level 1	*× *	*\checkmark *	*0.8380± 0.0453*	*10.37± 11.78*	*-0.0122*	0.962	*<0.001*
Single U-Net	*× *	*× *	*0.8356± 0.0479*	*15.21± 36.66*	*-0.0146*	0.971	*<0.001*

*p* values were obtained from paired *t* tests versus the full model with Bonferroni correction ($\alpha _{\textrm{corr}}=0.0167$, *k=3*). Level 1, SPADE fusion; Level 2, cross-attention fusion

## Discussion

This study presents DNQ-UNet, a hierarchical two-level fusion framework for few-shot domain adaptation in cervical cancer CTV segmentation. The method achieved effective target-domain adaptation with only 5–10 fine-tuning cases. The two-level design provides complementary adaptation mechanisms at different granularities: Level 1 uses spatially adaptive normalization for appearance alignment, whereas Level 2 uses cross-attention for semantic transfer [22–24]. This decoupled architecture allows adaptation to domain-specific style differences while preserving the second U-Net as the main fine segmentation engine.

The extended few-shot experiments showed a clear adaptation trajectory: DSC increased from 0.804 in the zero-shot setting to 0.818, 0.830, and 0.838 with 3, 5, and 10 fine-tuning cases, respectively. The 3-shot setting improved overlap but showed unstable boundary accuracy, with elevated HD95 and high sensitivity, indicating a tendency toward over-segmentation under extreme data scarcity. This instability was reduced in the 5-shot and 10-shot settings, supporting 10 cases as a more stable operating point when boundary accuracy is prioritized.

The added baseline experiments further support the sample efficiency of the proposed framework. Compared with full fine-tuning of nnU-Net V2, SwinUNETR, and MedNeXt, DNQ-UNet achieved the highest DSC at the 5-shot and 10-shot settings under the same independent target-domain test set. These comparisons do not imply exhaustive superiority over all possible few-shot segmentation algorithms; rather, they show that DNQ-UNet provides a practical 3D adaptation strategy that can be directly applied to volumetric pelvic CT without per-inference support prompts or additional auxiliary datasets. Existing few-shot medical segmentation approaches can be broadly divided into episodic support-set-based methods and pre-training/fine-tuning-based methods. DNQ-UNet follows the latter paradigm, which is more compatible with clinical radiotherapy deployment because the model can be adapted once using a small local cohort and then used as a fixed contour-initialization model.

The ablation study showed that both fusion levels contributed meaningfully. Level 1 (SPADE) produced the largest Cohen’s *d*, suggesting that appearance alignment yields the most consistent per-case benefit. This is plausible because inter-institutional differences in scanner-specific image appearance, acquisition protocol, and reconstruction setting are expressed strongly in low-level features [22]. Level 2 (cross-attention) also provided a measurable benefit, especially for semantically complex boundaries that require broader contextual reasoning [23, 24].

From a deployment perspective, the 5–10-case fine-tuning result is clinically attractive. The first U-Net serves as a compact prior extractor that can adapt to local style variations, while grouped differential learning rates protect the more general segmentation knowledge stored in the second U-Net. This design differs from approaches that adapt all parameters jointly [15–17] and may therefore be appealing in settings where only limited adaptation data and limited computational resources are available. In clinical practice, DNQ-UNet should be regarded as a locally adapted contour-initialization tool rather than a replacement for radiation oncologist review. A conventional source-domain model can also generate initial contours, but systematic domain-specific errors may require repeated manual correction after deployment to a new institution. The intended advantage of DNQ-UNet is to reduce the number of local annotated cases required for adaptation and to provide initial contours that better match local imaging characteristics and contouring conventions.

The domain-shift analysis further supports the need for local adaptation. Across five shared cases, 46 slices showed contouring discrepancies, with a maximum $1-\textrm{IoU}$ of 0.231; discrepancies were concentrated at the uterine fundus, parametrial margins, and cervicovaginal junction. Together with imaging-protocol differences between the GE source-domain scanner and the Philips target-domain scanner, these contouring-convention differences represent the main target-domain shift addressed by the SPADE appearance-alignment and cross-attention semantic-transfer modules.

This study has several limitations. First, training data were drawn from a single source institution and adaptation was evaluated on one target domain, so broader multi-center validation is still required. Second, the target-domain test set was relatively small (*n=20*), and the 3-shot setting showed unstable boundary accuracy, indicating that extremely low-data adaptation requires cautious validation. Third, the study did not directly benchmark performance against multiple human readers in the target domain or measure physician editing time, so claims regarding clinical workflow efficiency should be interpreted cautiously. Future work should therefore include larger multi-center validation, quality-assurance workflows for deployment, prospective assessment of editing time and clinical acceptability, and active-learning strategies to further reduce annotation burden.

## Conclusions

DNQ-UNet demonstrates that hierarchical two-level fusion can enable efficient few-shot domain adaptation for cervical cancer CTV segmentation. In this study, 5–10 annotated target-domain cases were sufficient to recover a substantial portion of the performance loss observed under zero-shot transfer. The framework provides a practical route toward reducing annotation burden and improving locally adapted contour initialization when deploying AI-assisted contouring systems in institutions with limited annotated target-domain data.

## Electronic Supplementary Material

Below is the link to the electronic supplementary material.

Supplementary Table S1. Source-domain sanity-check comparison between DNQ-UNet and nnU-Net V2 on the independent source-domain test set (n = 102).

## Data Availability

The data that support the findings of this study are available from the corresponding author upon reasonable request.
