# Supplementary material for: Dnq-unet: a two-level fusion framework for few-shot domain adaptation in cervical cancer CTV segmentation
Source: Radiol Phys Technol. 2026 Jun 3;19(3):998–1008. doi: 10.1007/s12194-026-01072-8 (PMC13546316; doi:10.1007/s12194-026-01072-8)
Supplement: Supplementary file 1 [file 12194_2026_1072_MOESM1_ESM.docx]

**Supplementary material**

*DNQ-UNet: A Two-Level Fusion Framework for Few-Shot Domain Adaptation in Cervical Cancer CTV Segmentation*

**Supplementary Table S1.** Source-domain sanity-check comparison between DNQ-UNet and nnU-Net V2 on the independent source-domain test set (n = 102).

| **Metric** | **DNQ-UNet** | **nnU-Net V2** | **Difference** | **p value** |
| --- | --- | --- | --- | --- |
| Dice (mean ± SD) | 0.8502 ± 0.0454 | 0.8439 ± 0.0510 | +0.0063 | <0.05 |
| IoU (mean ± SD) | 0.7421 ± 0.0660 | 0.7332 ± 0.0726 | +0.0089 | <0.05 |
| Sensitivity (mean ± SD) | 0.8599 ± 0.0648 | 0.8741 ± 0.0678 | -0.0142 | ns |
| Specificity (mean ± SD) | 0.9993 ± 0.0003 | 0.9992 ± 0.0004 | +0.0001 | ns |
| HD95 (mm) (mean ± SD) | 11.15 ± 26.37 | 10.32 ± 10.28 | +0.82 | ns |

ns, not significant. DNQ-UNet achieved better performance in 61 of 102 cases (59.8%).
